# Motor Development of Children in the Kurdistan Region of Iraq: Parent Survey

**DOI:** 10.3390/children11020162

**Published:** 2024-01-26

**Authors:** Francesca Policastro, Nizar Bakir Yahya, Alessandra Rossi, Giorgia Silli, Giovanni Galeoto, Nezar Ismet Taib

**Affiliations:** 1 Department of Medicine and Health Science, University of Trieste, 34127 Trieste, Italy; giorgia.silli@gmail.com; 2 College of Medicine, University of Duhok, Duhok 42001, Kurdistan Region, Iraq; nizar.yahya@uod.ac; 3 Italian Association for Solidarity Among People (AISPO) NGO, Duhok 42001, Kurdistan Region, Iraq; rossi.alessandra@hsr.it; 4 Department of Human Neurosciences, Sapienza University of Rome, Neuromed IRCCS, 00185 Rome, Italy; giovanni.galeoto@uniroma1.it; 5 Department of Medical Sciences, Child and Adolescent Psychiatry, Uppsala University, 75105 Uppsala, Sweden; nezar.tahib@neuro.uu.se

**Keywords:** motor milestones, child development, Kurdistan region of Iraq, socio-cultural context, environment

## Abstract

The actual literature highlights the importance of the socio-cultural context in the development of children. However, there is a lack of specific evidence about the middle East, especially regarding the development of Kurdish children who are living in a post-war scenario, in a country which is experiencing continuous instability due to the different crises. The main aim of this study is to identify the features of the motor development of Kurdish children according to parents’ opinion. A comparison with Italian children is provided as a Western example, which reflects data from the literature. In the study, 331 parents of Kurdish and Italian children aged between 3 and 7 years were involved. Parents filled the questionnaire at kindergartens, after providing consent. The questionnaire was conceptualized, designed, tested and provided ad hoc for this study; it focused on the timing of development, concerning major milestones like head control, sitting and standing-up. The questionnaire consists of 15 questions and has not been standardized yet. A logistic regression showed several differences between Kurdish and Italian children, like head control (*p* = 0.007) or the manipulation of big objects (*p* < 0.0001). These results identify the effect of the socio-cultural context and the impact of the growing environment of the child. Moreover, the results of this survey show the need for introducing different adapted, translated and validated assessment tools for motor development, considering differences related to the socio-cultural context.

## 1. Introduction

The present study was initiated during the implementation of an international cooperation project carried out by the international NGO Associazione Italiana Solidarietà per i Popoli (AISPO) in partnership with the Directorate General of Health in Duhok (Iraqi Kurdistan), funded by the Italian Agency for Development Cooperation and the World Health Organization (WHO). Since 2013, the AISPO has been supporting the improvement of children’s healthcare in the province of Duhok, Kurdistan Region of Iraq, involving clinical experts and consultants. While observing and dispensing the necessary assessment tools for motor development, experts from the AISPO and local staff set up this preliminary study.

Motor development is traditionally thought to follow a predictable and fixed sequence of milestones [1]. These milestones are considered the markers of a child’s development from infancy to childhood. The literature provides a range of ages for standardized motor developmental norms that can help in identifying delayed development and facilitate earlier interventions [2]. Nevertheless, most textbooks refer to a Western middle-class developmental pathway, not considering the culture and the environment [3]. Luckily, in the present literature, some studies about culture-related variations in motor development exist [4,5]. However, the actual literature does not provide validated tools for evaluating the motor development of babies and children in the Kurdish context. Taking this specific post-conflict socio-cultural context into account, the team of experts considered an investigation of the motor skills of Kurdish children necessary. For the preliminary study, we chose a parent-reported questionnaire, which is cheaper and saves time. For this, a motor development-related survey was set up and administered to Kurdish and Italian parents. The primary aim of this research is collecting parents’ reports about the motor development of their children. Through this tool, the experts aimed to deeply explore the motor development in the context of Kurdish culture in order to be more aware about and prepared for the best international, validated test or scale to choose for clinical examination of motor skills. We also administered the survey to an Italian population sample, which reflects the Western developmental pathway often described in textbooks [1,3].

The literature confirms the need for standardized assessment tools to evaluate children’s motor development in other cultures. Using the tools with normative data from different countries and cultures may lead to misinterpretation of the results, especially in labeling delayed or “early achieving” children [1]. The late evidence demonstrates that the cultural background is one of the strongest predictors of motor development, and all practitioners should be aware about the existing differences between contexts [6,7,8].

This paper presents results of the first part of a larger study, which also considers the cognitive and linguistic development of children in the Kurdish context for whom further data will be available in the future. The novelty of this study is the consideration of the motor skills of Kurdish children living in a post-war scenario, with social, economic and health issues due to the different crises that they are experiencing.

## 2. Methods

### 2.1. Study Design

This is an observational cross-sectional study on the motor development of children in the Kurdish context. We designed a survey for parents concerning the main motor developmental stages of the first years of life. A multi-step procedure was implemented with the following phases: questionnaire development conceptualization, design, testing and survey. The multi-step process was carried out during the summer of 2019; the collection of the data took place between October and December 2019, after obtaining ethical approval (15 October 2019).

### 2.2. Development Phase—Conceptualization

Conceptualization and operationalization were performed as the first phases for developing the questionnaire according to the methodological guidelines [9]. Before starting the operationalization phase, we performed a review of the existing literature to verify the concepts required, and an informal communication with experts and users. After this review, the survey concepts were established and translated into observable variables. The pertinent indicators were selected. The original draft was in English, and then a non-medical professional translator and an expert in the motor field performed the Kurdish translation and adaptation to the Kurdish context. Unclear Kurdish terms were edited into commonly used and understandable expressions. In the last step, two different experts in the motor field performed a back-translation into English and verified eventual inconsistencies with the original English draft. There were no difficulties in reconciling the back-translated version. A Kurdish native speaker senior pediatrician and a senior psychiatrist revised and proofread the final version.

A focus group was organized to verify the coherence between the concepts and the used indicators. An Italian physiotherapist guided the focus group as a moderator. A small number of people from the target population made up part of the focus group.

The aim of the focus group was to understand how potential respondents use and understand the general concepts and specific terminology, and if they consider the questions difficult or sensitive. Once the aims and the concepts were defined, the operational plan and a list of variables were created according to the expected output of the survey. The variables were separated into two groups: demographic variables and variables used for the survey concepts. 

The data collection mode was defined in this phase. The survey was self-administered by parents using a paper-and-pencil form. After that, researchers added the data into a database.

### 2.3. Development Phase—Design

During this phase, the data collection mode was defined. Moreover, the first draft of the questionnaire was created by considering the sequence of the thematic sections. Concrete questions were created and the questionnaire was implemented.

### 2.4. Development Phase—Testing

This phase is crucial to identify the problems for both respondents and interviewers, by considering the wording and content, order/context effects and visual design. The testing started after the full implementation of the draft questionnaire. Confusion with the meaning of the questions and misinterpretation of individual terms or concepts are usual wording problems, and could lead to missing data and frustration of the respondents. However, no wording problems emerged during this testing phase. 

### 2.5. The Questionnaire

AISPO’s experts and consultants created the questionnaire ad hoc for this project. 

The investigation focuses on the main early stages of motor development, concerning motor milestones from 0 to 6 years of age. The questionnaire recalls international validated scales, which were not completely applicable to the Kurdish context, such as the Bailey Scales of Infant Development [10], Development Coordination Disorder questionnaire [11], Wee-FIM [12] and Movement Assessment Battery for Children-2 (and its checklist) [13].

The questions focus on the timing of development, concerning head control, changing posture, sitting, crawling, standing up, walking, manipulation of big objects, manipulation of small objects, toileting, dressing, building 9-cube towers, scissoring, drawing, biking and shoe-tying. No subscales were included in the questionnaire. Each question was administered as a multiple choice and consisting of 4 possibilities; for each question, the choices were discussed between the experts involved after a deep, shared study of the actual literature 

The reliability and internal consistency of the questionnaire were assessed with the focus group in a field test with 22 Kurdish parents, which repeated the test twice after a week. Cronbach’s Alpha for reliability was 0.68, and Pearson’s correlation coefficient for internal consistency ranged from 0.44 to 1.

### 2.6. Survey—Data Collection Methods

The administration took place between October and December 2019 at 10 schools and 10 kindergartens in Duhok. Considering the different areas of the city, 5 schools from the poor/middle area and 5 schools from the richer area were chosen. The same proportion was respected for the kindergartens. The directors of the kindergartens approached the parents and gave them the appointment for participating in the study. After receiving the informed consent, six trained operators informed each potential participant about the strategy to answer the questionnaire. Approximately 15 min was needed to complete the questionnaire. During the self-report, the operators remained available to support the participants with any question. 

Between October and November 2019, in collaboration with the University of Trieste, the Italian version of the questionnaire was administered to 80 Italian parents, from the province of Trieste. Taking into account the consistency between the Italian data and the present literature on motor development, these data were collected as the control group. As part of the north-global countries, Italian children seemed to make up an appropriate group to compare the Kurdish children with.

### 2.7. Participants

For this study, 331 parents of Kurdish and Italian children aged between 3 and 7 years were recruited (Table 1). The only inclusion criterion to participate in the study was providing written consent. In order to have a representative sample, no other inclusion criteria were applied regarding the health status of the child.

### 2.8. Data Analysis

The data analysis was carried out using R and the package Rcmdr [14].

The main features of the sample were described appropriately using Fisher’s test and *t*-tests. The logistic regression was provided (with Wald’s correction) by grouping the sample considering the provenience. Statistical significance was considered at *p* < 0.05.

### 2.9. Ethical Considerations

According to the Declaration of Helsinki, this study was approved by the Ethics Committee of the Directorate of Health in Duhok with protocol number 15102019-7. Kurdish and Italian parents were enrolled on a voluntary basis. All participants were informed about the aims of the studies before starting the data collection. They were also informed that their data and all sensitive information were confidential and safely stored. The signed informed consents were obtained before starting the data collection. The participants had the right to withdraw from the study at any time.

## 3. Results

Table 1 summarizes the main features of the sample. The two groups of participants show two statistically significant differences related to age (*p*-value < 0.001 ***) and the number of children in the family (*p*-value < 0.001 ***).

### Survey Results

Table 2 summarizes the results of the logistic regression by grouping the sample considering the provenience. Italian participants are considered as the control group, reflecting the values of the literature.

No other variables were considered for the logistic regression because the aim of this study focused on the socio-cultural peculiarities of the motor development of Kurdish children.

Considering the significant *p*-values, Table 3 summarizes the results of the qualitative analysis.

## 4. Discussion

The socio-cultural context has an important impact on the motor development of children [3,8]. The lack of validation of tools is often an issue for international cooperation and humanitarian actions. The aim of this study was to preliminarily investigate the motor development of Kurdish children by comparing them with literature values. The created questionnaire permits for a preliminary assessment of the motor developmental stages according to parents’ report. By grouping the sample considering the provenience, the logistic regression provides statistically significant differences in the development of head control, ability to change position and sitting. According to the literature, most Kurdish (61.75%) and Italian (58.75%) parents affirm that head control develops between the third and sixth month. Nevertheless, 19.92% of Kurdish and 35.00% of Italian parents answered that their children develop this skill between the first and the third month. Moreover, differences regarding later development emerge: 3.98% of Kurdish, but no Italian parents, affirm that head control develops between the ninth and the twelfth month. Taking into account the distribution of the sample, according to parents’ report, Kurdish children seem to develop this skill a little later compared to data from the literature and the Italian group. Head control is itself a prerequisite needed to develop integrating vision, oral motor functions, trunk and arm control, and safe eating [15,16]. Early, at around one month of age, babies begin to show postural control responses in neck muscle activities. Delay and deficits of head control can interfere with subsequent motor milestones [17].

Different factors play an important role in head control. The literature shows that the time spent in prone position when a baby is awake has a direct relationship with the achievement of head control at three [18] and four [19] months. The WHO [2] confirms the importance of the prone position for motor development during the activity of the toddlers. In the Kurdish context, the prone position is still not accepted considering the risk of sudden infant death syndrome (SIDS) [1,20]. This attitude is causing children to experience the prone position later. Avoiding placement in the prone position decreases the possibility of the baby to develop antigravity movements with head and back. Prone positioning while awake promotes the acquisition and quality not only of prone skills, but also skills in other positions [15]. In fact, reduced tummy time could be one of the factors explaining the differences between the Kurdish and Italian answers about changing position, sitting and standing up. In fact, the potential benefits of tummy time include effects on an infant’s gross motor development [21]. However, the absence of public nurseries or daycare centers in Kurdistan might have an impact on the development of these motor abilities, comparing the increased enrollment rate in these structures from Western countries [22].

A significant statistical difference in the manipulation of big objects (like big blocks) exists, but for small objects (like small blocks), it does not, with a *p*-value = 0.05. Considering big objects, 58.75% of Italian parents report that their children start to use such objects between the sixth and tenth month; only 9.56% of Kurdish parents answered that their children started manipulating big objects at this age. This delay can be explained by many different factors. Literature highlights the importance of active playing for the development of milestones, even for motor ones [23]. Learning occurs when children actively engage in practical activities (such as playing) within a supportive social context. Object manipulation and play also emerge from early sensorimotor exploration, including the use of the mouth [24]. Through these steps, a child can learn the proprieties of the object itself and decide how to use it. On the other hand, in the Kurdistan context, children are often entertained with tablets, mobiles and television, even at very early stages [25]. Media use often encourages passivity and consumption of others’ creativity [22]. The WHO is suggesting avoiding the overuse of technological devices, and some awareness campaigns have been set up, but in Kurdistan, this practice is still ongoing. These activities are sedentary, do not engage the children and take away the time for real playing and learning. Nevertheless, the absence of nurseries and daycare centers is an issue to consider. Coherently with this result, the difference between Kurdish and Italian parents even emerges for the activity of building a nine-cube tower; according to parents’ answers, 65.00% of Italian children start to build a nine-cube tower at 2–3 years of age; at this age, only 9.16% of the Kurdish sample achieves this competence.

Considering the manipulation of small objects, the risk of choking might play an important role in the answers of the sample. In fact, in both cultures, the parents pay much attention not to propose small objects at the early stages. For this, a statistical difference between the two groups does not exist.

Scissoring and drawing are both manual dexterity skills which need a supportive social context to develop, especially in first steps. According to parents’ answers, a significant statistical difference between Kurdish and Italian children exists considering when they start to use scissors and draw. Nevertheless, attendance of a daycare center or nursery is a key point for the development of these skills, considered as necessary pre-school abilities [26].

Regarding biking, a statistical difference between Italian and Kurdish answers exists. In fact, the majority of Italian parents affirm (28.75%) that children start to independently bike between 3 and 4 years of age; on the contrary, 38.46% of Kurdish parents affirm that their children start between 5 and 6 years of age. The option “not all the children have a bike” could have an impact on this difference; in fact, 29.48% of Kurdish parents and 12.50% of Italians have chosen this answer. Highlighting another important cultural issue is necessary in order to understand this difference: in Kurdistan, young girls are frequently not involved in physical activities like biking, or there are no accessible facilities, especially for girls.

There are even important implications about self-care to consider. In fact, regarding toileting, dressing and shoe-tying, parents’ ‘answers don’ not show any difference between the two groups.

## 5. Strengths and Limitations of the Study

The actual literature reveals that this is the first study which considers and analyzes the motor development of Kurdish children by taking into account parents’ opinions. This study has a different strength, including the possibility of utilizing these results for future epidemiological studies, and future adaptation and validation of clinical tools.

Some limitations must also be considered. No sample size was estimated before the administration of the questionnaires. Illiterate Kurdish parents were included in the study because they were supported by the six operators to answer the questionnaire. However, involving this group of parents, we did not consider the possible effect of educational qualifications and socioeconomic disadvantage in the answers. The children’s gender was not considered during data analysis.

## 6. Conclusions

The actual study highlights the peculiarities of the motor development of Kurdish children according to their parents’ opinions. Compared to Italian children who reflect the literature’s data, differences emerge regarding the following milestones: head control, changing posture, sitting, standing up, manipulation of big objects, nine-cube tower, scissoring, drawing and biking. The present questionnaire gives important insights into the cultural differences which can have an impact on motor development. The created tool was appropriated in order to clarify and deeply assess the motor development of children in the Kurdish culture by considering that the data emerge from the parents themselves. AISPO’s experts and the local staff are already considering the present survey while choosing tests and scales to administer to children and designing follow-up actions.

## Figures and Tables

**Table 1:** Description of the sample.

	Group	Italian	Kurdish	*p*-Value
Gender, n (%)	Male	20 (25%)	54 (21.5%)	0.539
	Female	60 (75%)	197 (78.5%)	
Age, years (mean, sd)		41.2 (5.7)	37.0 (7.7)	<0.001 ***
Number of children in the family (mean, sd)		1.7 (0.7)	3.7 (1.6)	<0.001 ***
Educational level, n (%)	No School	0 (0%)	34 (15.1%)	<0.001 ***
	Primary School	0 (0%)	41 (18.2%)	
	Middle School	8 (10.1%)	22 (9.8%)	
	High School	27 (34.2%)	101 (44.9%)	
	Degree	44 (55.7%)	27 (12%)	

n = quantity, sd = standard deviation; Note—significance level *p* < 0.001 ***, *p* > 0.05 not significant.

**Table 2:** Logistic regression considering the provenience (Kurdish or Italian).

Question	Odds Ratio	Confidence Interval	*p*-Value
Head control	2.15	1.23–3.75	0.007 **
Changing posture	2.44	1.45–4.13	<0.001 ***
Sitting	2.90	1.52–5.57	0.001 **
Crawling	2.03	0.85–4.84	0.11
Standing up	3.86	1.14–13.00	0.03 *
Walking	3.23	1.01–10.30	0.05
Manipulation big objects	13.00	7.08–24.00	<0.001 ***
Manipulation small objects	1.72	1.00–2.94	0.05
Toileting	<0.001	0-inf	0.99
Dressing	2.19	0.35–13.30	0.39
Nine-cube tower	20.30	10.70–38.50	<0.001 ***
Scissoring	6.23	3.03–12.80	<0.001 ***
Drawing	3.84	1.25–11.80	0.02 *
Biking	3.05	1.61–5.77	<0.001 ***
Shoe-tying	0.54	0.26–1.14	0.10

Note—significance levels: *p* < 0.001 *** extremely significant, 0.001 < *p* < 0.01 ** very significant, 0.01 < *p* < 0.05 * significant, *p* > 0.05 not significant.

**Table 3:** Qualitative analysis of the statistically significant variables for comparison of the two groups.

	Age Band (%)				
Head control	0–3 months	3–6 months	6–9 months	9–12 months	Not answered
Kurdish	19.92%	61.75%	12.35%	3.98%	1.99%
Italian	35.00%	58.75%	5.00%	0.00%	1.25%
Changing posture	4–6 months	6–8 months	8–10 months	After 10 months	Not answered
Kurdish	40.24%	48.61%	8.76%	0.80%	1.59%
Italian	61.25%	32.50%	2.50%	1.25%	2.50%
Sitting	4–6 months	6–8 months	8–10 months	After 10 months	Not answered
Kurdish	10.36%	45.02%	31.87%	10.36%	2.39%
Italian	25.50%	53.75%	16.25%	2.50%	2.50%
Standing up	4–8 months	8–12 months	12–15 months	After 15 months	Not answered
Kurdish	1.99%	31.87%	55.38%	7.57%	3.19%
Italian	7.50%	60.00%	30.00%	2.50%	0.00%
Big objects	6–10 months	10–16 months	16–20 months	After 20 months	Not answered
Kurdish	9.56%	32.67%	29.48%	26.29%	1.99%
Italian	58.75%	36.25%	3.75%	0.00%	1.25%
Nine-cube tower	2–3 years	3–4 years	4–5 years	5–6 years	Not answered
Kurdish	9.16%	24.30%	37.85%	27.09%	1.59%
Italian	65.00%	27.50%	3.75%	0.00%	3.75%
Scissoring	2–3 years	3–4 years	4–5 years	5–6 years	Not answered
Kurdish	5.98%	17.93%	18.33%	53.39%	4.38%
Italian	27.50%	48.75%	15.00%	2.50%	6.25%
Drawing	1–2 years	2–3 years	3–4 years	4–5 years	Not answered
Kurdish	2.39%	11.55%	29.69%	55.38%	1.99%
Italian	8.75%	46.25%	38.75%	6.25%	0.00%
Biking	3–4 years	4–5 years	5–6 years	Not using the bike	Not answered
Kurdish	14.34%	17.53%	34.26%	29.48%	1.99%
Italian	28.75%	21.25%	12.50%	12.50%	25.00%

## Data Availability

The data presented in this study are available on request from the corresponding author. The data are not publicly available due to privacy or ethical restrictions.
